# Effect of electroacupuncture on discomfort during gastroscopy: a study protocol for a randomized controlled trial

**DOI:** 10.1186/s13063-022-06165-4

**Published:** 2022-04-27

**Authors:** Binyu Yu, Philippa Jemma Hazlewood, Xuan Yin, Shanshan Li, Hongyu Yue, Kun Xu, Shifen Xu, Yiqun Mi

**Affiliations:** Shanghai Municipal Hospital of Traditional Chinese Medicine, Shanghai University of Traditional Chinese Medicine, Shanghai, China

**Keywords:** Electroacupuncture, Gastroscopy, Discomfort, Randomized controlled trial

## Abstract

**Background:**

Gastroscopy procedures are frequently performed under general sedation to minimize discomfort. Patients who refuse a sedative injection may experience more discomfort and adverse reactions such as pain and nausea. These instances reduce patient compliance and willingness to participate in future procedures. Acupuncture has been shown to have an anti-nausea and analgesic effect; however, there is limited data available that demonstrates the efficacy of acupuncture when applied before gastroscopy.

**Methods:**

A total of 60 participants will be randomly assigned to the electroacupuncture (EA) group and the sham electroacupuncture (SEA) group at a ratio of 1:1. Acupuncture treatment will be performed before gastroscopy for a duration of 30 min. All patients will complete detailed questionnaires at 30 min and 7 days post-procedure to record the severity of their symptoms. The primary outcome will be the average of 4 standard visual analogue scale (VAS) scores in the categories of nausea, vomiting, throat discomfort, and agitation as reported by the patient. The secondary outcomes will be patient’s anxiety level as recorded by the 6-item short form of the State-Trait Anxiety Inventory (STAI-S6) and Amsterdam Pre-Operative Anxiety and Information Scale (APAIS), preference in a future endoscopy, pulse oxygen saturation (SpO2), heart rate (HR), and blood pressure (BP). Anxiety scales will be assessed before and after acupuncture; others will be completed at 30 min and 7 days post-procedure. The duration of the gastroscopy and the number of biopsies will be recorded after operation.

**Discussion:**

This randomized controlled trial will explore the feasibility of the further clinical application of electroacupuncture for the improvement of patient discomfort during gastroscopy without systemic sedation.

**Trial registration:**

ChiCTR2000040726. This trial has been approved by the Ethics Committee of Shanghai Municipal Hospital of Traditional Chinese Medicine (2020SHL-KY-11). Registration date 12 August 2020.

## Administrative information

**Table:** 

Title {1}	Effect of Electroacupuncture on Discomfort during Gastroscopy: a study protocol for a randomized controlled trial
Trial registration {2a and 2b}	This trial has been approved by the Ethics Committee of Shanghai Municipal Hospital of Traditional Chinese Medicine (2020SHL-KY-11) and is registered as ChiCTR2000040726.
Protocol version {3}	version 1.0
Funding {4}	This research is supported by the Shanghai Hospital Development Center (grant SHDC2020CR3015A)
Author details{5a}	1.Shanghai Municipal Hospital of Traditional Chinese Medicine, Shanghai University of Traditional Chinese Medicine, Shanghai, China
Name and contact information for the trial sponsor {5b}	Contact name: Clinical Research Plan of SHDC Address: 2,Kangding Road, Shanghai, 200041, China Phone code: 021-96886
Role of sponsor {5c}	The sponsor played no part in study design; and will play no part in the collection, management, analysis, and interpretation of data; writing of the report; and the decision to submit the report for publication.

## Introduction

### Background and rationale {6a}

Upper gastrointestinal endoscopy (gastroscopy) is one of the most common diagnostic and therapeutic methods for assessing upper gastrointestinal diseases [1]. Gastroscopy is the first choice for clinical examination of upper gastrointestinal diseases because physicians can directly observe the inspection site and perform pathological biopsy and ligation on the diseased tissue in real time [2]. Due to the aging population and modern dietary changes, the average number of gastroscopies performed annually has sharply increased over the past few decades [3].

The strong stimulation from gastroscope insertion can often induce a gag reflex, which causes throat discomfort and nausea. Unmedicated patients also experience anxiety, increased blood pressure, and tachycardia during gastroscopy procedure. To curtail these symptoms, gastroscopy under general anesthesia is widely used. However, the use of anesthesia is costly and associated with inhibited cognitive function and mental decline. Moreover, the post procedural impairment affects a patients’ ability to drive and return to work immediately afterwards [4]. That is why approximately half of Chinese gastroscopy patients decline general anesthesia during gastroscopy [5, 6]. We therefore need to identify a safe and efficacy method to reduce adverse events during gastroscopy and offer patients a palatable alternative to sedation. Acupuncture analgesia has its unique advantages. Clinical trials have confirmed that preoperative acupuncture can reduce anxiety, decrease the use of anesthetic drugs, relieve nausea and vomiting, and shorten hospital stays [5–8]. Acupuncture during the perioperative period can help restore intestinal function and reduce postoperative pain [9, 10]. Studies have reported acupuncture analgesia as a successful alternative to pharmacological sedation during gastroscopy; however, the actual benefits still require further studies to corroborate [11, 12]. Therefore, high-quality randomized clinical trials are needed to re-validate previous findings and develop a clinical trial of electroacupuncture to reduce patients’ discomfort during standard gastroscopy.

### Objectives {7}

We designed a rigorous randomized controlled clinical trial using a single-center, patient- and assessor-blinded placebo-controlled research method to evaluate the impact of electroacupuncture (EA) on the discomfort of patients undergoing gastroscopy. We aim to observe whether EA can reduce the discomfort of patients during gastroscopy; the results will help provide evidence for whether EA is an effective therapy to reduce patient discomfort during general gastroscopy.

### Trial design {8}

**Fig. 1:**
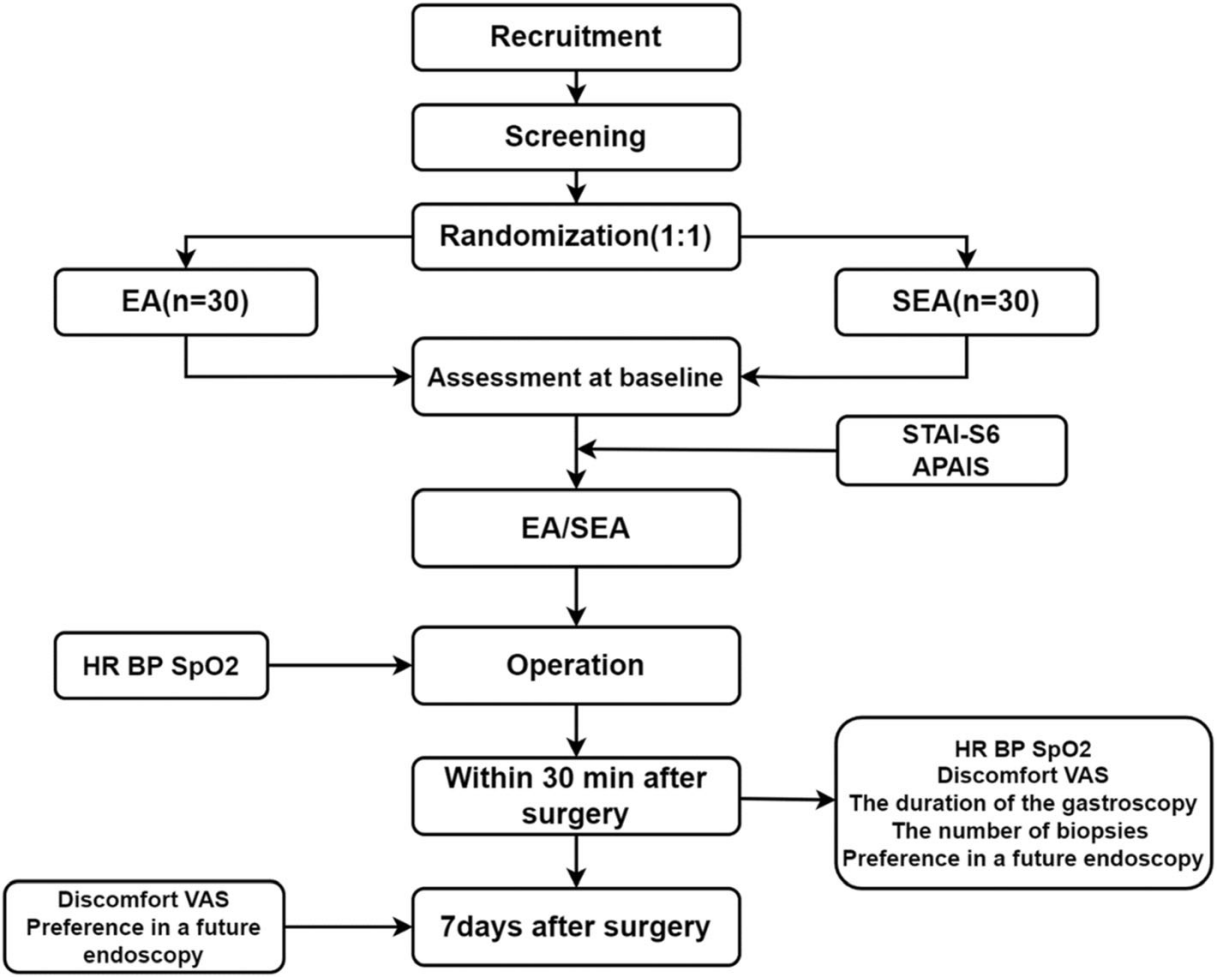
Flowchart of this study

This RCT is a single-site randomized placebo-controlled trial. It will be implemented in Shanghai Municipal Hospital of Traditional Chinese Medicine. All eligible patients will be randomly assigned to the electroacupuncture group (EA) or the sham electroacupuncture (SEA) group in a 1:1 allocation ratio. All patients must sign the informed consent form before they are enrolled in the trial. The research process flow chart is detailed in Fig. 1. In this trial, the Consolidated Standards of Reporting Trials (CONSORT) [13] and acupuncture reporting guidelines, Standards for Reporting Interventions in Clinical Trials of Acupuncture (STRICTA) [14], will be followed.

## Methods: participants, interventions, and outcomes

### Study setting {9}

All eligible participants will be recruited through the outpatient clinic or inpatient, hospital-based WeChat advertising, and posters in the Shanghai Municipal Hospital.

### Eligibility criteria {10}

#### Inclusion criteria

Patients who meet the following conditions will be included:

- 18–70 years old, male or female
- Patients willing to undergo gastroscopy with local anesthesia
- Patients with clear consciousness and can answer the questions, understand the scales, and complete the assessment
- Patients who agree to participate and sign a written informed consent

#### Exclusion criteria

Patients who meet any of the following conditions will be excluded:

- Patients with mental disorders or severe cognitive impairments who cannot participate in cooperation
- Patients with a history of bleeding disorders or who currently use warfarin or low-molecular-weight heparin
- Patients who cannot receive acupuncture for reasons such as infections around the acupuncture points or allergy to acupuncture needles
- Patients who have received acupuncture treatment in the past 6 months
- Participants in other clinical trials that may interfere with the primary endpoint

### Who will take informed consent? {26a}

All participants will be recruited by advertising through online and offline recruitment channels in Shanghai Municipal Hospital. People who need gastroscopy and are interested in participating can contact the researcher by calling or adding the WeChat. We will evaluate according to the inclusion and exclusion criteria. All participants will be clearly informed about the potential benefits and risks of the trial before they sign the informed consent.

### Additional consent provisions for collection and use of participant data and biological specimens {26b}

Not applicable.

### Interventions

#### Explanation for the choice of comparators {6b}

In this trial, all participants will receive the electroacupuncture or sham electroacupuncture before the gastroscopy. We will adopt the superficial acupuncture in the non-acupoints method. It has the advantages of good masking effect, not easily perceived by the patient, and a higher feasibility of operation.

#### Intervention description {11a}

All participants will receive the electroacupuncture or sham electroacupuncture before the gastroscopy. They will then undergo standard upper gastrointestinal endoscopy procedures. Patients in both groups will be discharged within 1 h after the endoscopy, which includes a 30-min observation period, a preference for future endoscopy assessment and blinding assessment. The acupuncture methods are described in Table 1. To maintain blinding, patients will be asked to wear an eye-patch and receive treatment in a supine position in an isolated space with limited communication with the acupuncturist. The acupuncturist will disinfect the patient’s skin with 75% alcohol cotton balls before treatment. Acupuncture treatment will last 30 min. After removing the needle, researchers will use a clean cotton ball to compress the acupoint to prevent bleeding. A total of 8 points will be used. Table 2 summarizes the acupoints and rationale.

**Table 1:** Treatment methods of electroacupuncture and acupoints

	EA group	SEA group
Acupoints	Hegu (LI4), Neiguan (PC6), Zusanli (ST36), Liangqiu (ST34)	Non-acupoints as shown in Table 3
Depth of insertion	10 mm:LI4 PC6;30 mm:ST36 ST34	Superficial acupuncture
Needle type	Steel needles (Wuxi Jiajian Medical Co. Ltd. Wuxi, China)	The same as EA group
Needle sensation	With de-qi sensation	Without de-qi sensation
Electric stimulation	Two pairs of needles:	Two pairs of needles:
	LI4–ST36 (bilaterally)	LI4–ST36 (bilaterally)
	Connected to CMNS6-1 (Wuxi Jiajian Medical Device CO., China)	Connected to CMNS6-1 (Wuxi Jiajian Medical Device CO., China)
	Deliver continuous wave-type low-frequency 2 Hz and current of 2 mA	No electrical current delivered

*EA* electroacupuncture, *SEA* sham electroacupuncture

**Table 2:** Acupoint selection and rationale based on traditional Chinese medicine

Acupoint	Location	Traditional Chinese medicine indication	Suggested technique
LI4 (Hegu)	Dorsum of hand, at the level of the midpoint of the second metacarpal bone, between the first and second metacarpal bones	Relieve visceral pain	Needle perpendicular,0.5–1.0 cun
PC6 (Neiguan)	Palmar aspect of the forearm, between the tendons, 2 cun away from the transverse crease of the wrist	Reduce nausea and stomach pain	Needle perpendicular,0.5–1.5 cun
ST36 (Zusanli )	Antero-lateral leg, 1 middle-finger breadth next to the anterior crest of tibia, 3 cun under the depression lateral to the patellar ligament	Reduce nausea and stomach pain	Needle perpendicular,1–2 cun
ST34 (Liangqiu)	2 cun above the bottom of the patella, on the line between the anterior superior iliac spine and the lateral end of the bottom of the patella	Relieve stomach pain	Needle perpendicular,1–1.2 cun

##### The electroacupuncture group

Participants in the treatment group will receive EA treatment. The standard acupuncture method will be applied at 8 acupoints: bilateral Hegu (LI4), Neiguan (PC6), Zusanli (ST36), and Liangqiu (ST34). All acupoints were selected based on literature, clinical experience, and acupuncture textbooks. The acupuncture needles will be disposable sterile needles made of stainless steel (0.25 × 40 mm and 0.30 × 40 mm; Jia Jian, China). After insertion, needles will be manipulated using the lifting-thrusting and twirling technique. A pair of electroacupuncture apparatus (CMNS6-1, Wuxi Jiajian Medical Device CO, China) will be connected to 2 pairs of needles (LI4, ST36 bilaterally) to deliver a continuous wave, low frequency (2 Hz), and current of 2 mA.

##### The sham electroacupuncture group

**Fig. 2:**
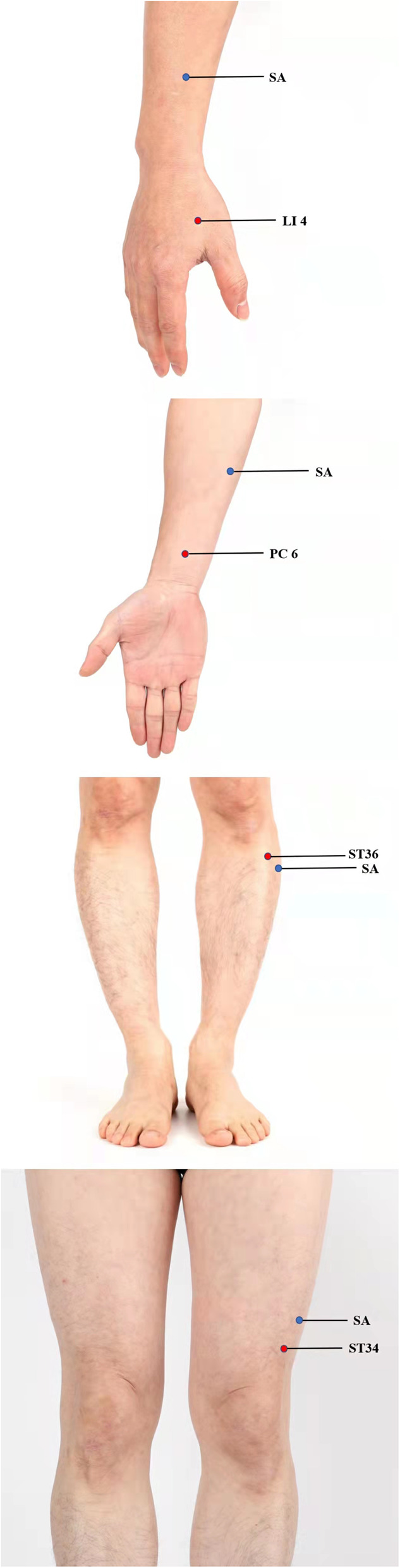
Superficial acupuncture on the non-acupoints

In the control group, we will use the same acupuncture needles as used in the EA group but repurposed as placebo needles. The acupuncturist will perform superficial acupuncture on the non-acupoints detailed in Table 3 and shown in Fig. 2. All of the needles will be inserted using a needle tube to a superficial depth (2–3 mm) without invoking the sensation of “De-qi.” The electroacupuncture apparatus will be connected to 2 pairs of needles (LI4, ST36 bilaterally) for 30 min, but all indicators will be set to “0” to ensure no electrical current is delivered.

**Table 3:** Non-acupoints positioning

Acupoints	Non-acupoints
Hegu (LI4)	1 cun beside Zhigou (SJ6), between the sanjiao channel and the large intestine channel
Neiguan (PC6)	In the middle of the medial epicondyle of the humerus and the styloid process of ulna
Zusanli (ST36)	3 cun below Yanglingquan (GB34), between the gallbladder and bladder meridian
Liangqiu (ST34)	3 cun below Zhongdu (GB32), between the stomach meridian and the gallbladder meridian

#### Criteria for discontinuing or modifying allocated interventions {11b}

When acupuncture-related serious adverse reactions occur during the experiment or there is a failure to complete the study for any other reasons, we will record the details in a report for the early termination of the study.

#### Strategies to improve adherence to interventions {11c}

At the beginning of the trial, participants will be again informed of the experiment procedure. They can report their personal feelings at any time during the study and we will give corresponding feedback as appropriate to increase the interactivity and improve adherence to interventions.

#### Relevant concomitant care permitted or prohibited during the trial {11d}

Patients cannot eat 8 h before gastroscopy to ensure a fasting state.

#### Provisions for post-trial care {30}

At the end of the study, we will provide a subsidy to each subject.

### Outcomes {12}

#### Primary outcome

A visual analogue scale (VAS) will be used to evaluate the patient’s level of discomfort, which is a line segment from 0 to 10, with 0 indicating low and 10 indicating high discomfort. All patients will complete 4 detailed VAS at 30 min. The questionnaire will evaluate 4 areas of discomfort including nausea and vomiting, throat discomfort, bucking, and agitation. The primary outcome is the average score of the 4 VAS.

#### Secondary outcomes

- The patient’s level of discomfort at 7 days post-procedure by using VAS. The four VAS including nausea and vomiting, throat discomfort, bucking, and agitation will present separately as secondary outcomes at 30 min and 7 days post-procedure.
- Cardiac-respiratory assessment

All patients’ pulse oxygen saturation (SpO2), heart rate (HR), and blood pressure (BP) measurements will be recorded prior to, during, and post-procedure. Then, each patient’s “double product” (DP) will be calculated at each point of the procedure, based on the systolic blood pressure (SBP) and heart rate (SBP×HR). DP is a useful prognostic tool for patients with poor cardiac function during exercise and rest, reflecting myocardial oxygen consumption and cardiac output during treadmill testing. A value of DP greater than 15,000 indicates cardiovascular stress. It is accepted that changes in cardiac output from baseline (preoperative) will reflect the level of stress experienced by patients undergoing the gastroscopy.

- Anxiety level

A six-item short form of the State-Trait Anxiety Inventory (STAI-S6) and Amsterdam Pre-operative Anxiety and Information Scale (APAIS) will assess the degree of preoperative anxiety, postoperative anxiety, and 7 days post-procedure. These questionnaires are quick and simple for participants to complete, which is advantageous in time-restricted studies.

- Preference in a future endoscopy

The questionnaire will ask patients whether they have undergone gastroscopy before, and then ask, “Do you have interest in taking acupuncture treatment for to avoid the post-operative discomfort before next gastroscopy?”

- Safety assessment

Before participants sign the informed consent form, they will be informed that acupuncture clinical trials may have potential adverse events such as bleeding, hematomas, fainting, bruising, ecchymoma, and possible infection. All details of the adverse event will be recorded by the patients and doctors. Adverse events (AE) are rated as 1 (mild), 2 (moderate), or 3 (severe or medically significant). All details of AEs will be recorded in the case report form (CRF). At the end of the trial, we will analyze the impact of all events.

- Blinding success assessment

After the gastroscopy procedure, researchers will evaluate the success of the blinding by asking participants the following question: “what kind of treatment do you think you have received?” The options are traditional acupuncture treatment, superficial acupuncture treatment, and uncertain. If participants do not choose “uncertain,” then the researcher will question the patient further for specific reasons.

- Related to gastroscopy

The duration of the gastroscopy and the number of biopsies will be recorded after operation.

### Participant timeline {13}

The timing of intervention and data collection is detailed in Table 4.

**Table 4:** Schedule of enrolment, intervention, and assessments

Timepoint	Screening	Baseline	Intervention		Operation			Follow-up
			Before	After	Before	During	30 min after endoscopy	Day 7
**Basic information**								
Informed consent	X							
Inclusion/exclusion	X	X						
**Interventions**								
EA			X	X				
SEA			X	X				
**Assessments**								
**Primary outcome**								
Discomfort VAS							X	
**Secondary outcomes**								
Discomfort VAS							X	X
STAI-S6			X	X				
APAIS			X	X				
HR BP and SpO2					X	X	X	
Preference in a future endoscopy							X	X
The duration of the gastroscopy							X	
The number of biopsies							X	
**Others**								
Adverse events			X	X	X	X	X	X
Patient satisfaction							X	
Success of blinding							X	

*EA* electroacupuncture, *SEA* sham electroacupuncture, *VAS* visual analogue scales, *STAI-S6* six-item short form of the State-Trait Anxiety Inventory, *APAIS* Amsterdam Pre-operative Anxiety and Information Scale, *HR* heart rate, *BP* blood pressure, *SpO2* oxygen saturation

### Sample size {14}

Hypotheses: We hope to provide conclusive evidence to test the hypothesis that acupuncture is superior than sham acupuncture in reducing the discomfort of patients during gastroscopy. In our pilot study, the discomfort VAS assessment at 30 min post-procedure was 4.8 (SD, 1.76) in the EA group and 6.3 (SD, 1.53) in the SEA group. We used PASS software to calculate that 50 patients would be needed to provide a 90% power to detect a difference of discomfort VAS in 2 groups at a 2-sided significance level of 5%. If we assume that 20% of patients would be lost to follow-up, 60 would need to be enrolled.

### Recruitment {15}

Participants of the study will be recruited through the outpatient clinic or inpatient, hospital-based WeChat advertising, and posters in the Shanghai Municipal Hospital. Any patients who are willing to participate will be screened by telephone or on site and consented for the study. There is one specialist who will answer the phone to answer questions from these kinds of disposed patients.

Research assistants will screen the patients, obtain written informed consent, and assign them to either the EA or SEA group, to receive a traditional acupuncture or an acupuncture-like simulation treatment.

## Assignment of interventions: allocation

### Sequence generation {16a}

In this trial, we plan to use SPSS24.0 software to generate a random number table, which divides eligible participants into the EA group or SEA group with a 1:1 ratio.

### Concealment mechanism {16b}

A random distribution card will be made and sealed with an opaque envelope. Participants will be informed that they have an equal chance of being assigned to the EA or SEA group before the trial.

### Implementation {16c}

An independent researcher, blinded to the study protocol, will generate the allocation sequence. Another researcher will give the opaque envelope to the acupuncturist according to the timing sequence of participant registration for the trial. Then, the acupuncturist will open the envelope to perform the corresponding acupuncture operation.

## Assignment of interventions: blinding

### Who will be blinded {17a}

This is a patient-assessor-blinded study. To ensure that participants are blind, they will be treated in an isolated space and be required to wear blindfolds. Only the acupuncturist who performs the treatment will know the group assignment at the time of treatment. Gastroenterologists, data analysts, and statisticians will remain blinded.

### Procedure for unblinding if needed {17b}

Unmasking is not needed.

## Data collection and management

### Plans for assessment and collection of outcomes {18a}

We will record the evaluation results in the CRFs in a timely fashion.

### Plans to promote participant retention and complete follow-up {18b}

Patients will come to the hospital 1 week after gastroscopy to complete the final questionnaires. We will follow up and debrief patients at this time point. At the end, patients will receive a transportation subsidy.

### Data management {19}

Patient characteristics will be recorded in the CRFs and stored in the researcher’s office. The clinical trial management platform ResMan will be used to manage the raw data. The raw data will be collected by assessors who are blinded to the group assignment and repeated input methods will be used to ensure that the entered data is correct. Before the platform is officially launched, it will be tested and the users will be trained. After the platform is officially launched, the database will be locked with a password, which will only be known by relevant personnel. The clinical director will oversee the work of the clinical trial center at least once a month.

### Confidentiality {27}

All the documents and materials related to this will be kept strictly confidential. Only once the trial is finished will the principal investigator distribute the data to the third parties. We will protect the privacy of participants’ personal medical information as required by law. The staff in this study is also bound by this agreement.

### Plans for collection, laboratory evaluation, and storage of biological specimens for genetic or molecular analysis in this trial/future use {33}

Not applicable. This trial does not have biological specimens.

## Statistical methods

### Statistical methods for primary and secondary outcomes {20a}

The independent statistician is responsible for the statistical analysis with spss24.0 software. Patient data that falls off will not be included in the analysis. In the statistical analysis, measurement data between the two groups will be analyzed with the *t*-test and rank-sum test, while categorical data will be analyzed with *χ*^2^ test. Data will be recorded as mean ± standard deviation or median (first quartile, third quartile). The significance level used for statistical analysis will be two-sided with confidence intervals at the 95% level.

### Interim analyses {21b}

Not applicable.

### Methods for additional analyses (e.g., subgroup analyses) {20b}

Not applicable.

### Methods in analysis to handle protocol non-adherence and any statistical methods in analysis to handle protocol non-adherence and any statistical methods to handle missing data {20c}

We perform statistical analysis on complete case.

### Plans to give access to the full protocol, participant-level data, and statistical code {31c}

All data sharing will be conducted in accordance with the regulatory requirements.

## Oversight and monitoring

### Composition of the coordinating center and trial steering committee {5d}

In order to control the quality of the clinical trial, the whole process of the trial will be conducted under the supervision of a qualified clinical trial expert in Shanghai Municipal Hospital of Traditional Chinese Medicine. The Clinical Research Center for Drugs of Shanghai University of Traditional Chinese Medicine will conduct the data monitoring. When problems occur in the trial, the center has the right to make the final decision to terminate the trial if necessary. Any changes will be notified in writing to all participants in the trial after approval by the ethics committee. The principle investigator will be fully responsible for conducting the trial and will make any final decisions.

### Adverse event reporting and harms {22}

We will record any adverse events such as bleeding, hematomas, fainting, bruising, ecchymoma, and possible infection.

### Frequency and plans for auditing trial conduct {23}

We will audit every 3 months; any modification that may have an impact on the study and potential benefit to patients or affect patient safety, including changes of the study objective, study design, patient population, sample size, study procedure, or serious adverse events, will be reported to the Committee. This will be decided by jointly with the monitoring committee, the Clinical Research Center of Drugs, and approved by the Ethics Committee.

### Plans for communicating important protocol amendments to relevant parties (e.g., trial participants, ethical committees) {25}

When the trial protocol is modified during the trial, we will conduct a discussion group composed of statisticians, acupuncturists, gastroenterologists, etc. After collective discussion, the next step of the trial plan will be drawn up and the clinical plan updated on the clinical trial registration website.

### Dissemination plans {31a}

The publication of the outcomes of this study will provide baseline data. The outcomes may be presented at conferences, symposiums, university classes, etc., if applicable.

## Discussion

Acupuncture therapy has been effectively used in clinical practice for relieving symptoms caused by clinical surgery. For example, Wang et al. [15] ADDIN show that acupuncture may reduce the side effect of vomiting during gastroscopy by adjusting the function of vegetative nerves, regulating gastrointestinal tract function, and reducing the concentration of blood 5-HT. Taras I U [16] found that auricular acupuncture (AA) is a promising alternative treatment for situational anxiety. Andrzejowski JC [17] found that acupuncture at the EX-HN3 point reduces preoperative anxiety levels in patients awaiting neurosurgery. However, these existing literatures have inevitable deficits: differing acupuncture intervention method such as using ear acupuncture or intra-dermal needles and a lack of a uniform standard for the setting of intervention time. Some papers have reported that when applied before gastroscopy acupuncture has no apparent differences compared to sham acupuncture [18, 19]. So, in this trial, we used the study by Liu et al. [20], which has been widely used in clinical practice as a successful implementation of sham acupuncture, to rule out the placebo effect of acupuncture in a rigorous way. Studies have shown that superficial acupuncture in non-acupoints method has the advantages of providing a good masking effect and being not easily perceived by the patient and has a higher feasibility of operation. One clinical trial of acupuncture treatment for pain found that the superficial acupuncture at non-acupoints method is an effective way to control for placebo [21]. By designing this RCT, we aim to contribute better evidence for the efficacy of electroacupuncture in reducing discomfort during gastroscopy through an improved design and methods.

For patients, surgical procedures performed in hospitals are a strong stressor and can lead to an anxiety response. Excessive anxiety can affect immune mechanisms through the release of corticosteroids and may be associated with abnormal hemodynamics as a consequence of endocrine changes and activation of the central nervous system [22, 23]. A high level of preoperative anxiety will also reduce patients’ satisfaction with the procedure and increase the risk of postoperative nausea and vomiting [24, 25]. So, in this trial, we will focus on preoperative anxiety. The STAI-S6 and APAIS are reliable means of assessing patient anxiety [26, 27]. By using these questionnaires, we can quickly and accurately assess patients’ anxiety level in a few minutes.

Previous findings have shown that gastroscopy may bring a series of complications to patients with hypertension, such as myocardial infarction, cardiac arrest, and other complications [28]. Some research has shown that in patients older than 45 years, the risk of cardiovascular disease doubles with an increase in systolic blood pressure of 20 mmHg or a diastolic blood pressure increase of 10 mmHg [29, 30]. So, in our study, we will monitor BP before, during, and after gastroscopy, then analyze the correlation between DP and the primary outcome of VAS score to determine whether EA can reduce stress placed on the heart, and thus benefit elderly people with cardiovascular related diseases who need gastroscopy.

There are several limitations in this study. First of all, we did not limit any comorbidities that patients may have. Patients who meet our inclusion criteria and need upper gastrointestinal endoscopy according to the assessment of a gastroenterologist can be included in this trial. Some comorbidities like pharyngitis might increase sensitivity and discomfort when the gastroscope passes through the pharynx. Therefore, we cannot precisely determine the impact of comorbid diseases on the level of anxiety during gastroscopy. Secondly, based on the previous pilot research, the sample size of this RCT include 60 patients, which is still a small-scale study involving a small sample, but the data obtained in this study will support the sample size calculation of future multi-center, large-scale clinical trials. We hope, in the future, the data will have guiding significance for subsequent clinical trials related to acupuncture intervention in gastroscopy. Finally, it is inevitable for the acupuncturist to know the grouping of patients. We hope that this limitation will be offset by the fact that the acupuncturist does not participate in the evaluation of the questionnaire and that the patients are subjected to rigorous blinding practices.

We aim to design a standard and rigorous RCT to reduce discomfort during gastroscopy by acupuncture treatment. We believe acupuncture will increase patient compliance and ease the gastroscopy-related burden on public health services. With positive results, our research will serve as a new standard of care for patients hoping to forego systemic sedation during gastroscopy procedures.

### Trial status

We started recruiting participants in February 2021 and the recruitment is expected to end late 2021.

## Abbreviations

VAS: Visual analogue scales
STAI-S6: The 6-item short form of the State-Trait Anxiety Inventory
APAIS: Amsterdam Pre-Operative Anxiety and Information Scale
SpO2: Pulse oxygen saturation
HR: Heart rate
BP: Blood pressure
STRICTA: Standards for Reporting Interventions in Clinical Trials of Acupuncture
